# Readmission for Acute Exacerbation within 30 Days of Discharge Is Associated with a Subsequent Progressive Increase in Mortality Risk in COPD Patients: A Long-Term Observational Study

**DOI:** 10.1371/journal.pone.0150737

**Published:** 2016-03-04

**Authors:** Mónica Guerrero, Ernesto Crisafulli, Adamantia Liapikou, Arturo Huerta, Albert Gabarrús, Alfredo Chetta, Nestor Soler, Antoni Torres

**Affiliations:** 1 Respiratory Department, Clinic Institute of Respiratory (ICR), Hospital Clinic of Barcelona - Institut d'Investigacions Biomèdiques Agusti Pi i Sunyer (IDIBAPS) - CIBERES - University of Barcelona (UB), Barcelona, Spain; 2 Department of Clinical and Experimental Medicine, Respiratory Disease and Lung Function Unit, University of Parma, Parma, Italy; 3 6^th^ Respiratory Department, Sotiria Hospital, Athens, Greece; University of Athens, GREECE

## Abstract

**Background and Objective:**

Twenty per cent of chronic obstructive pulmonary disease (COPD) patients are readmitted for acute exacerbation (AECOPD) within 30 days of discharge. The prognostic significance of early readmission is not fully understood. The objective of our study was to estimate the mortality risk associated with readmission for acute exacerbation within 30 days of discharge in COPD patients.

**Methods:**

The cohort (n = 378) was divided into patients readmitted (n = 68) and not readmitted (n = 310) within 30 days of discharge. Clinical, laboratory, microbiological, and severity data were evaluated at admission and during hospital stay, and mortality data were recorded at four time points during follow-up: 30 days, 6 months, 1 year and 3 years.

**Results:**

Patients readmitted within 30 days had poorer lung function, worse dyspnea perception and higher clinical severity. Two or more prior AECOPD (HR, 2.47; 95% CI, 1.51–4.05) was the only variable independently associated with 30-day readmission. The mortality risk during the follow-up period showed a progressive increase in patients readmitted within 30 days in comparison to patients not readmitted; moreover, 30-day readmission was an independent risk factor for mortality at 1 year (HR, 2.48; 95% CI, 1.10–5.59). In patients readmitted within 30 days, the estimated absolute increase in the mortality risk was 4% at 30 days (number needed to harm NNH, 25), 17% at 6-months (NNH, 6), 19% at 1-year (NNH, 6) and 24% at 3 years (NNH, 5).

**Conclusion:**

In conclusion a readmission for AECOPD within 30 days is associated with a progressive increased long-term risk of death.

## Introduction

In the natural course of Chronic Obstructive Pulmonary Disease (COPD), many patients experience acute exacerbations (AECOPD), characterized by an increase in systemic inflammatory activity [1] and by a deterioration of their respiratory signs and symptoms [2]. The rate of AECOPD becomes more frequent across disease severity [3]. However, some patients have an increased risk of acute recurrence or relapse following the initial episode [4], linked to increased susceptibility to exacerbations [5,6].

In general, COPD patients with higher rate of acute exacerbations have a greater annual decline in lung function [7] and present poorer clinical outcomes [5,6,8]. With regard to prognosis, previous studies have demonstrated that AECOPD represents an independent risk of death due to all causes [8], regardless of the level of lung function impairment [9]. In one long-term evaluation, the mortality risk was strictly related to the number of AECOPD episodes [10].

Hospital readmissions in COPD patients are known to have clinical and socioeconomic consequences, especially if they are necessary soon after discharge (for example, within 30 days) [11]. Although the majority of readmissions in COPD patients are not respiratory-related [12], around 20% are readmitted due to AECOPD within 30 days after discharge [13]. Although several predictors have been identified [14,15] and certain interventions have been proposed [16] there is not enough evidence at present to recommend a single disease-specific approach to address the problem [12,16].

To date, no studies have evaluated readmission for AECOPD within 30 days as a prognostic factor in COPD patients. The aim of this study was therefore to estimate, in both short and long-term follow-up periods, the risk of death due to all causes in patients presenting an acute exacerbation within 30 days of discharge and requiring re-hospitalization.

## Material and Methods

### Study Design and Patients Selection

This observational study was conducted at a university hospital in Spain (Hospital Clinic, Barcelona) over a period of 64 months between May 2009 and September 2014. The sampling method was systematic and all AECOPD patients admitted to the hospital’s Respiratory Department were considered for the study. The patients included had to meet criteria for COPD according to the GOLD guidelines [17]. Spirometry was performed in the stable phase of the disease and at least six months prior to hospital admission. Patients with a smoking history of 10 pack-years were considered as positive smokers.

The study protocol was approved by the Hospital Research and Ethics Committee (CEIC 2008/4106) and conducted in accordance with good clinical practices and the declaration of Helsinki. Written informed consent was obtained from all the patients enrolled.

### Definitions

A worsening of the respiratory symptoms compared with preceding days which required a change in home care medication was used as the criterion for defining AECOPD [2,17]. The severity of the exacerbation was based on the respiratory symptoms/signs and the presence of potential indications for hospitalization [17].

A follow-up visit at 30 days from discharge after the index hospitalization confirmed readmission, defined as a readmission due to a new occurrence of symptoms/signs of AECOPD, using the same criteria [2]. Patients readmitted for reasons other than AECOPD were not considered.

### Exclusion Criteria

At hospitalization index and at readmission to hospital, the exclusion criteria concerned patients with a documented history of concomitant chronic respiratory condition (asthma and bronchiectasis), patients with a suspected underlying malignancy, and patients in whom community-acquired pneumonia (CAP) and acute heart failure were identified clinically and by means of chest x-ray.

### Microbiological Sample Collection

Sputum from patients was obtained from spontaneous cough on the first day of admission to hospital; an adequate sample, containing a count of more than 25 leukocytes and less than 10 epithelial cells per field, was processed using Gram stain and sputum culture. Inhalation of a 5% hypertonic saline solution for 5 to 10 minutes, delivered via a nebulizer device, was used to obtain induced sputum production when spontaneous sputum samples were not obtainable.

### Clinical Measurements and Outcomes

Data on demographic variables, body mass index (BMI), smoking habit, presence of co-morbidities measured by Charlson Index, prevalence of ischemic heart disease and diabetes, baseline dyspnea grade as per modified medical research council (mMRC), COPD severity score measured by a questionnaire COPDSS, use of long-term oxygen therapy (LTOT) and use of domiciliary medications (inhaled bronchodilators as short-acting β_2_ agonist [SABA], long-acting β_2_ agonist [LABA], and anticholinergics, inhaled corticosteroids) were recorded on admission to hospital. Characteristics of exacerbations occurring in the preceding year were also recorded.

Vital signs (body temperature, respiratory and heart rate, systolic and diastolic blood pressure) were assessed at admission. Gas analysis variables (pH, partial arterial carbon dioxide pressure [PaCO_2_], the ratio of partial arterial oxygen pressure to the fraction of inspired oxygen [PaO_2_/FiO_2_], serum bicarbonate [HCO_3_^-^], base excess [BE]), and systemic inflammatory variables (leukocytes, haematocrit, haemoglobin, C-reactive protein [CRP], glucose, and creatinine were recorded at admission and at day 3.

Variables relating to clinical progression included length of stay in hospital (LOS), use of noninvasive and invasive mechanical ventilation (NIMV and IMV), and intensive care unit (ICU) admission recorded during the initial hospitalization. Data on prognosis (cumulative number of deaths for all-causes, time to death) were recorded in a follow-up period of 30 days, 6 months, 1 year and 3 years; centralized registries were used to identify the date of death.

### Statistical Analysis

Table 1 shows number of patients (%) for categorical variables, and medians (1^st^ quartile; 3^rd^ quartile) for continuous variables with non-normal distribution or means ±SD for those with normal distribution. Categorical variables were compared using the χ^2^ test or the Fisher exact test and continuous variables with the *t* test or the nonparametric Mann-Whitney test. Univariate and multivariate Cox proportional hazard regression analyses [18] were performed to identify variables associated with 30-day readmission (the dependent variable). The variables included in the univariate analysis were: age, gender, BMI, smoking habit, respiratory rate, heart rate, temperature, systolic and diastolic blood pressure, number of exacerbations, number of exacerbations requiring hospitalization, number of days prior to exacerbation, COPD-SS, Charlson index, mMRC dyspnea score, FEV_1_, FVC, CRP at admission, glucose at admission, creatinine at admission, leukocytes at admission, haematocrit at admission, haemoglobin at admission, pH at admission, PaCO_2_ at admission, PaO_2_/FiO_2_ at admission, ICU admission, NIMV, IMV, and LOS.

**Table 1:** General characteristics of study cohort.

Variables	Cohort of patients (N = 378)	Patients with 30-day readmission* (N = 68)	Patients without 30-day readmission* (N = 310)	p value
Age, years	71.4 ± 10	72.2 ± 10.6	71.2 ± 9.9	0.475
Male	318 (84)	60 (88)	258 (83)	0.306
BMI, kg/m^2^	27.4 ± 5.4	27.3 ± 5.2	27.4 ± 5.4	0.915
Smoking habit: Current/ No or Former,%	39/61	25/75	42/58	**0.009**
FEV_1_,% predicted †	44.2 ± 16.9	39.2 ± 14	45.3 ± 17.3	**0.005**
FEV_1_/FVC †	48.6 ± 14.4	46.8 ± 14.8	49 ± 14.4	0.299
GOLD stage: A/B/C/D,%	3/30/48/19	0/17/60/23	3/33/45/19	**0.046**
LTOT	104 (28)	32 (47)	72 (23)	**<0.001**
mMRC baseline dyspnea grade	2 (1; 3)	3 (2; 3)	2 (1; 3)	**0.013**
COPDSS severity questionnaire	13 (8; 19)	18 (13; 21)	12 (7; 18)	**<0.001**
Charlson index	2 (1; 3)	2 (1; 4)	2 (1; 3)	0.130
Ischemic heart disease	26 (7)	2 (3)	24 (8)	0.059
Diabetes	91 (24)	20 (29)	71 (23)	0.262
No of exacerbations ‡	0 (0; 1)	1 (0; 2)	0 (0; 1)	**<0.001**
No of patients with ≥2 exacerbations ‡	83 (22)	26 (39)	57 (19)	**<0.001**
No of exacerbations requiring hospitalization ‡	0 (0; 1)	0 (0; 1)	0 (0; 1)	**0.044**
No of patients with ≥1 exacerbations requiring hospitalization ‡	114 (31)	27 (40)	87 (28)	0.056
No of previous days until exacerbation	4 (2; 7)	3 (2; 7)	4 (2; 7)	**0.020**
*Use of home care medications*				
Salbutamol only	12 (4)	2 (3)	10 (4)	>0.99
Anticholinergic only	18 (5)	4 (6)	14 (5)	0.772
LABA + Anticholinergic	4 (1)	0 (0)	4 (2)	0.584
LABA + ICS	9 (3)	1 (1)	8 (3)	0.691
Anticholinergic + ICS	5 (2)	1 (1)	4 (2)	>0.99
LABA + Anticholinergic + ICS	109 (33)	16 (24)	93 (36)	0.059

Data are shown as number of patients (%), means ± SD or medians (1^st^ quartile; 3^rd^ quartile), unless otherwise stated. Percentages are calculated on non-missing data.

*Abbreviations*: AECOPD: chronic obstructive pulmonary disease exacerbation; BMI, body mass index; FEV_1_, forced expiratory volume in the 1^st^ second; FVC, forced vital capacity; LTOT, long-term oxygen therapy; mMRC, modified medical research council; COPDSS, COPD severity score questionnaire; LABA, long-acting β_2_ agonist; ICS, inhaled corticosteroids.

* Since index hospitalization.

^†^ Data evaluated at least 6 months prior to index hospitalization.

^‡^ In a period of 1 year prior to index hospitalization.

LABA includes salmeterol, formoterol and indacaterol; Anticholinergic includes ipratropium and tiotropium; and ICS includes budesonide and fluticasone.

Univariate and multivariate Cox proportional hazard regression analyses were also performed to identify variables associated with 1-year mortality (dependent variable). The variables included in this univariate analysis were the ones mentioned above, plus 30-day readmission. Variables that showed a significant result (p<0.1) were included in the corresponding multivariate Cox proportional hazard regression backward stepwise model. Variables that correlated strongly (r>|±0.3|) were excluded from the multivariate analyses. Unadjusted and adjusted hazard ratios (HR) and 95% confidence intervals (CI) were calculated. Time-to-event variables (30-day, 6-month, 1 year and 3-year mortality) were analyzed by means of Kaplan-Meier survival curves (a Gehan-Breslow-Wilcoxon test was applied, because this test emphasizes early differences [19]). Patients who were lost to follow-up were censored in the survival analysis; moreover, in order to define potential survival bias, an analysis comparing patients considered for the study and those lost to follow-up after one year was performed. We also calculated the number needed to harm (NNH) [20] for the analyses involving 30-day readmission. This statistic represents the number of patients who would need to be readmitted for one death to occur. All reported p values were two-sided and were not adjusted for multiple comparisons. A p value less than 0.05 was considered significant. All analyses were performed with IBM SPSS Statistics 20.0 (Armonk, New York, USA).

## Results

### Patient Characteristics

**Fig 1:**
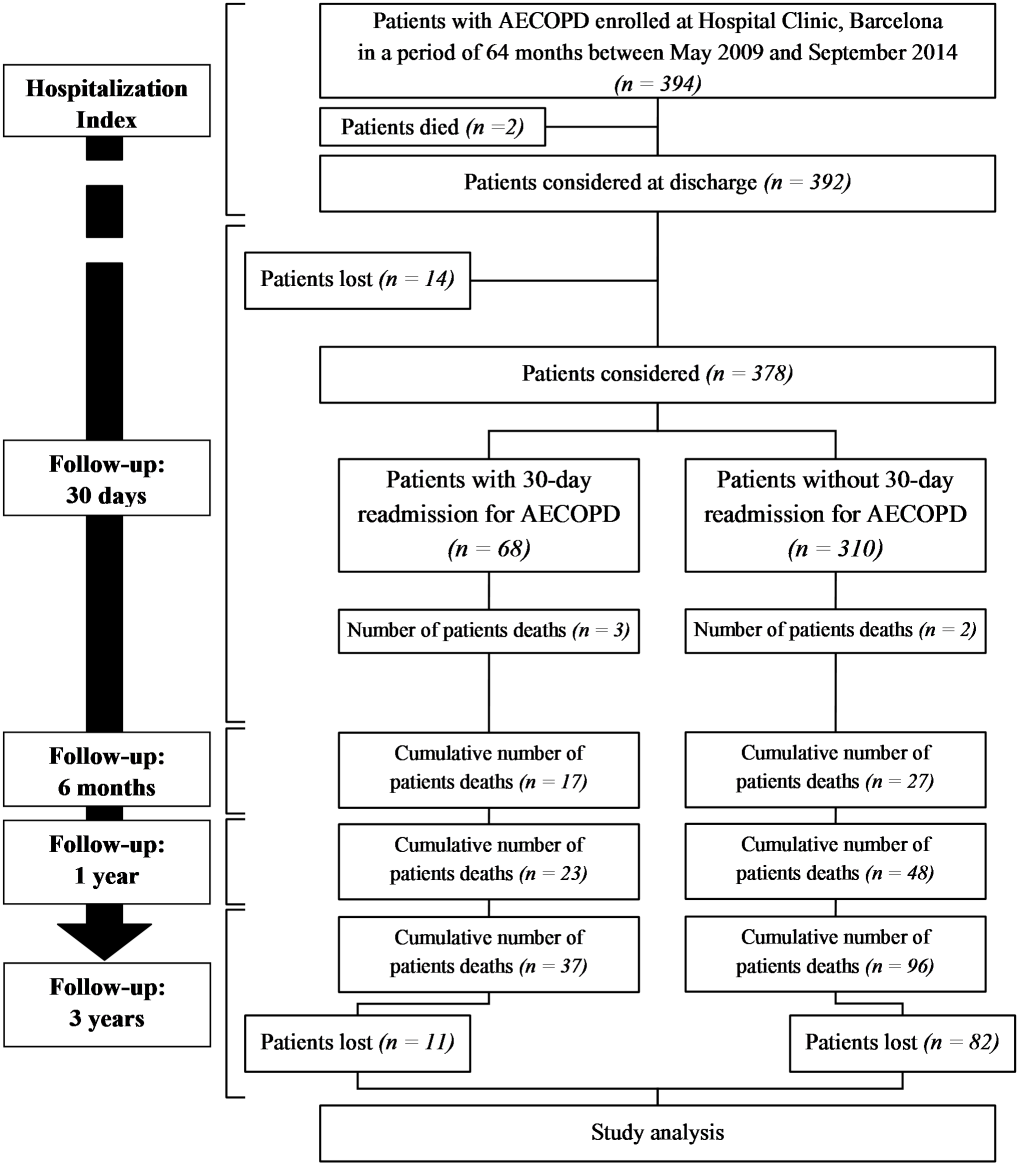
Study flow diagram. Abbreviations: AECOPD: chronic obstructive pulmonary disease exacerbation.

In total, 378 consecutive patients (84% men) with a mean±SD age of 71±10 years were admitted to hospital for an AECOPD. Of these, 68 patients (18%) had a new AECOPD that required hospitalization in the 30 days after discharge. Table 1 compares the characteristics of the patients enrolled in the study and divided according to readmission/non-readmission within 30 days, and Fig 1 shows the study flow diagram.

Early readmitted patients presented greater severity according to functional, symptomatic and questionnaire-reported characteristics of COPD than patients without 30-day readmission. Moreover, readmitted patients more frequent required LTOT and had a significant history of exacerbations in the previous year, also requiring hospitalization. No significant differences were found for age, sex, BMI, presence of co-morbidities or use of home care medication.

### Clinical and Microbiological Variables

With regard to clinical (Table 2) and microbiological (Table 3) variables recorded at admission and during the clinical course, patients with and without 30-day readmission showed differences only in terms of pH at day 3 (with lower values in readmitted patients), PaCO_2_ at admission (higher values), and haematocrit at day 3 (lower values). The other variables presented similar values in the two groups.

**Table 2:** Clinical and laboratory variables recorded at admission and during clinical course of index hospitalization.

Variables	Cohort of patients (N = 378)	Patients with 30-day readmission (N = 68)	Patients without 30-day readmission (N = 310)	p value
*Vital signs*				
Respiratory rate, b/min	24 (20; 28)	24 (20; 28)	24 (20; 28)	0.952
Heart rate, b/min	93 (82; 105)	91.5 (80; 104)	94 (83; 105)	0.296
Temperature, °C	36.3 (36; 36.9)	36 (36; 36.6)	36.4 (36; 37)	0.085
SBP, mmHg	139 (123; 156)	138 (123; 158)	139 (126; 151)	0.842
DBP, mmHg	75.5 (68; 86)	76 (68; 87)	73 (68; 84)	0.096
*Gas analysis and systemic inflammatory variables*				
pH at admission	7.39 (7.34; 7.43)	7.38 (7.32; 7.43)	7.39 (7.34; 7.43)	0.121
pH at day 3	7.41 (7.38; 7.44)	7.38 (7.35; 7.41)	7.42 (7.39; 7.44)	**0.002**
PaCO_2_ at admission, mmHg	47.3 (38.6; 58.3)	51.0 (40.3; 65.6)	46.0 (38.5; 57.6)	**0.048**
PaCO_2_ at day 3, mmHg	48 (41.3; 54.4)	50.9 (41.1; 62)	47.4 (41.3; 53)	0.271
PaO_2_/FiO_2_ at admission, mmHg	262 (219; 315)	258 (226; 317)	263 (218; 314)	0.987
PaO_2_/FiO_2_ at day 3, mmHg	280 (251; 316)	280 (246; 312)	280 (256; 319)	0.517
HCO_3_ at admission, mmol/L	28 (24.7; 32.2)	29 (24.5; 34.2)	27.9 (24.7; 31.6)	0.369
HCO_3_ at day 3, mmol/L	30 (27; 34.1)	31 (26.8; 34.9)	29.8 (27; 33.4)	0.612
BE at admission, mmol/L	2.3 (-0.2; 5.6)	2.4 (-0.9; 6.8)	2.3 (-0.1; 5.2)	0.958
BE at day 3, mmol/L	4.6 (2.2; 7.5)	5.1 (1.5; 8.8)	4.5 (2.6; 7.5)	0.977
Leucocytes at admission, x 10^9^/l	10.4 (7.7; 14)	9.8 (8.1; 14.6)	10.4 (7.7; 14)	0.913
Leucocytes at day 3, x 10^9^/l	10.6 (8.2; 13.2)	10.4 (7; 13.3)	10.7 (8.3; 13.1)	0.442
Haematocrit at admission,%	43 (40; 47)	42.5 (40; 45.5)	44 (39; 47)	0.140
Haematocrit at day 3,%	40 (37; 44)	39.5 (36; 42.5)	41 (37; 45)	**0.045**
Haemoglobin at admission, g/L	139 (126; 152)	136 (125.5; 147.5)	140 (126; 153)	0.142
Haemoglobin at day 3, g/L	131 (118; 142)	126 (118.5; 136.5)	132 (118; 143)	0.067
C-reactive protein at admission, mg/dL	3.8 (1.3; 10.4)	3.2 (1.4; 8)	3.9 (1.3; 10.5)	0.272
C-reactive protein at day 3, mg/dL	1.2 (0.4; 3.6)	1.8 (0.4; 4.3)	1.1 (0.4; 3.4)	0.366
Glucose at admission, mg/dL	125 (107; 159)	125.5 (104; 165.5)	124 (107; 155)	0.933
Glucose at day 3, mg/dL	119 (97; 157)	126 (103.5; 162.5)	116 (96; 154)	0.064
Creatinine at admission, mg/dL	0.9 (0.8; 1.1)	0.9 (0.8; 1.2)	0.9 (0.8; 1.1)	0.624
Creatinine at day 3, mg/dL	0.9 (0.7; 1.2)	0.9 (0.8; 1.2)	0.9 (0.7; 1.1)	0.681
No of patients using systemic corticosteroids *(Methylprednisolone)*	342 (92)	65 (97)	277 (91)	0.092
No of patients using antibiotics	311 (88)	58 (89)	253 (87)	0.660
Duration of antibiotic treatment, days	7 (5; 10)	7 (5; 10)	7 (5; 10)	0.964
Classes of antibiotics used				
Penicillins *(Amoxicillin*, *Amoxicillin/Clavulanate)*	53 (18)	10 (17)	43 (18)	0.945
Fluoroquinolones *(Ciprofloxacin*, *Moxifloxacin*, *Levofloxacin)*	156 (52)	28 (48)	128 (52)	0.567
Macrolides *(Azithromycin*, *Clarithromycin)*	6 (2)	2 (3)	4 (2)	0.325
Cefalosporins *(Ceftriaxone*, *Cefotaxime*, *Cefuroxime and Cefepime)*	10 (3)	4 (7)	6 (2)	0.090
Carbapenems *(Meropenem)*	1 (0.3)	0 (0)	1 (0.4)	>0.99

Data are shown as number of patients (%) or median (1^st^ quartile; 3^rd^ quartile), unless otherwise stated. Percentages are calculated on non-missing data.

*Abbreviations*: SBP and DBP indicate systolic and diastolic blood pressure respectively; PaCO_2_, partial arterial carbon dioxide pressure; PaO_2_/FiO_2_, ratio of partial arterial oxygen pressure to the fraction of inspired oxygen; HCO_3_, serum bicarbonate; BE, base excess.

**Table 3:** Microbiological variables.

	Patients with a 30-day readmission (N = 68)	Patients without a 30-day readmission (N = 310)	p value
Patients with positive cultures in sputum	17 (25)	62 (20)	0.358
Polymicrobial etiology	1 (6)	6 (10)	>0.99
*Streptococcus spp*.	3 (18)	14 (23)	>0.99
*Pseudomonas aeruginosa*	7 (41)	14 (23)	0.135
*Streptococcus pneumoniae*	1 (6)	6 (10)	>0.99
*Moraxella catarrhalis*	1 (6)	1 (2)	0.386
*Haemophilus influenzae*	0 (0)	12 (19)	0.060
*Staphylococcus spp*	2 (12)	2 (3)	0.201
*Serratia*	1 (6)	0 (0)	0.215
*Pasteurella*	1 (6)	1 (2)	0.386
*Mycobacterium no-TBC*	0 (0)	1 (2)	>0.99
*Aspergilus*	0 (0)	2 (3)	>0.99
*Candida spp*	0 (0)	2 (3)	>0.99
Patients positive for Influenza A/H_1_N_1_ in naso-pharyngeal swab *	7 (21)	20 (13)	0.279
*Influenza B virus*	2 (29)	0 (0)	0.060
*Respiratory syncytial virus*	2 (29)	2 (10)	0.269
*Rhinovirus*	1 (14)	6 (30)	0.633
*Parainfluenza virus type 1*	1 (14)	2 (10)	>0.99
*Parainfluenza virus type 3*	0 (0)	2 (10)	>0.99
*Parainfluenza virus type 4*	0 (0)	1 (5)	>0.99

Data are shown as number of patients (%).

* Percentages are calculated on total patients with nasopharyngeal swab in each group (N = 33 and N = 150 in the groups of patients with and without 30-day readmission respectively).

### Predictors of 30-Day Readmission Identified in Univariate and Multivariate Analyses

Several variables were significantly associated with 30-day readmission in the univariate Cox regression analyses (Table 4). In the multivariate analysis, a history of ≥2 exacerbations in the previous year (HR, 2.47; 95% CI, 1.51 to 4.05) was the only variable independently associated with 30-day readmission.

**Table 4:** Significant univariate and multivariate Cox regression analyses predicting the probability of a 30-day readmission.

Variable	Univariate			Multivariate		
	HR	95% CI	p value	HR	95% CI	p value
Age ≥70 y	1.72	1.01–2.92	0.045	-	-	-
Smoking habit *			0.026	-	-	-
No	1	-	-	-	-	-
Current	0.43	0.13–1.46	0.17	-	-	-
Former	0.90	0.28–2.90	0.87	-	-	-
Temperature (+1°C)	0.74	0.53–1.03	0.072	-	-	-
DBP (+1 mmHg)	0.98	0.96–1.00	0.056	-	-	-
No. of exacerbations ≥2 †	2.47	1.51–4.05	<0.001	2.47	1.51–4.05	<0.001
No. of previous days until exacerbation (+1 days)	0.93	0.87–0.99	0.017	-	-	-
COPD-SS ≥15 ‡	2.68	1.62–4.45	<0.001	-	-	-
mMRC dyspnea score ≥ 2 ‡	2.81	1.29–6.12	0.009	-	-	-
FEV_1_ <50% §	2.57	1.30–5.09	0.007	-	-	-
Haematocrit (+1%) ‡	0.96	0.92–1.00	0.068	-	-	-
Haemoglobin (+1 g/L) ‡	0.99	0.98–1.00	0.099	-	-	-
pH (+0.1 units) ‡	0.73	0.54–0.98	0.033	-	-	-
PaCO_2_ (+1 mmHg) ‡	1.02	1.00–1.03	0.009	-	-	-
LOS (+1 day)	1.04	1.01–1.07	0.003	-	-	-

* The p-value corresponds to differences between the three groups (non-smoker, current smoker, or former smoker).

^†^ In a period of 1 year prior to index hospitalization.

^‡^ Evaluated at admission for index hospitalization index.

^§^ Data evaluated at least 6 months prior to index hospitalization.

*Abbreviations*: see Tables 1, 2 and 5.

### Outcomes

**Fig 2:**
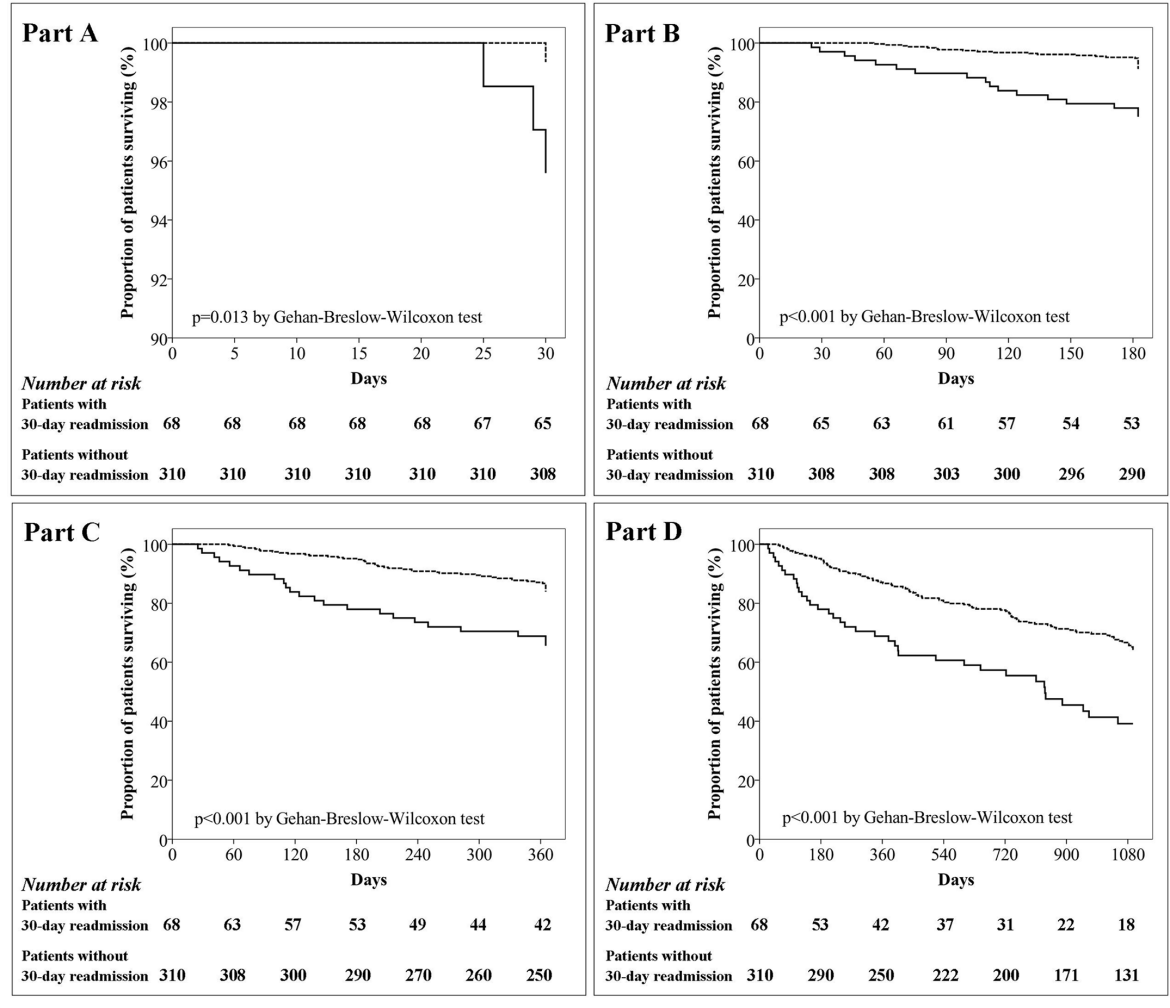
Kaplan-Meier survival curves in the follow-up period: at 30 days (part A), 6 months (part B), 1 year (part C), and 3 years (part D) from discharge after initial hospitalization. Continuous and dashed lines represent patients with and without 30-day readmission for AECOPD respectively.

Patients with a 30-day readmission had a longer median hospital stay than patients not readmitted (9 [6.5; 13] *vs*. 7 [5; 10] days, p = 0.003); no significant differences were found between the groups with regard to the use of NIMV and IMV or for ICU admission. The overall mortality after 30 days differed between readmitted and non-readmitted patients (5% *vs*. 1%, p = 0.045). Mortality evaluated after the follow-up periods of 6 months, 1 year and 3 years was also higher in the group of patients readmitted within 30 days (27% *vs*. 10%, p<0.001; 37% *vs*. 17%, p = 0.001; and 67% *vs*. 43% respectively, p = 0.001) and their periods of survival were shorter (109 *vs*. 162 days, p = 0.021; 124 *vs*. 209 days, p = 0.014; 250 *vs*. 445 days respectively, p = 0.023) (Table 5). In patients readmitted within 30 days, the estimated absolute increase in mortality risk was 4% at 30 days (NNH, 25; 95% CI, 8–243), 17% at 6 months (NNH, 6; 95% CI, 3–14), 19% at 1 year (NNH, 6; 95% CI, 3–13) and 24% at 3 years (NNH, 5; 95% CI, 3–11) (Table 6). All patients were followed up for survival. Before the corresponding follow-up period, only the data for the patients who were lost to follow-up were censored (on the date when they were last known to be alive). The comparison of general characteristics between patients followed for 1 year and lost (Table 7) did not show any significant variables potentially influencing the risk of death. Kaplan-Meier survival curves depicting death rates as a function of 30-day readmission are shown in Fig 2. In the four Kaplan Meier curves obtained at different periods of follow-up, the risk of death in patients readmitted within 30 days was higher than in patients not readmitted (Gehan-Breslow-Wilcoxon test p = 0.013, p<0.001, p<0.001, and p<0.001 in the follow-up at 30 days [Fig 2, part A], 6 months [Fig 2, part B], 1 year [Fig 2, part C] and 3 years [Fig 2, part D] respectively).

**Table 5:** Study Outcomes.

	Cohort of patients (N = 378)	Patients with 30-day readmission (N = 68)	Patients without 30-day readmission (N = 310)	p value
LOS, days	8.7 ± 5.6 8 (6; 10)	10.4 ± 6.7 9 (6.5; 13)	8.3 ± 5.3 7 (5; 10)	**0.003**
NIMV	82 (22)	17 (25)	65 (21)	0.473
IMV	14 (4)	3 (4)	11 (4)	0.724
ICU admission	45 (12)	8 (12)	37 (12)	>0.99
30-day mortality	5 (1)	3 (5)	2 (1)	**0.045**
Time to death (days)	28.8 ± 2.2 30 (29; 30)	28 ± 2.7 29 (25; 30)	30 ± 0 30 (30; 30)	0.20
6-month mortality	44 (13)	17 (27)	27 (10)	**<0.001**
Time to death (days)	124.5 ± 52.6 127 (78; 182.5)	101.2 ± 52.6 109 (56; 139)	139.1 ± 47.9 162 (86; 182.5)	**0.021**
1-year mortality	71 (21)	23 (37)	48 (17)	**0.001**
Time to death (days)	204.3 ± 109.4 198 (109; 313)	157 ± 106.9 124 (66; 237)	223.9 ± 104.4 209 (143; 332)	**0.014**
3-year mortality *	133 (48)	37 (67)	96 (43)	**0.001**
Time to death (days)	470.5 ± 324.4 406 (189; 739)	382 ± 326.6 250 (111; 648)	504.6 ± 318.7 445 (209; 748.5)	**0.023**

Data are shown as number of patients (%), mean ± SD or median (1^st^ quartile; 3^rd^ quartile), unless otherwise stated. Percentages are calculated on non-missing data.

*Abbreviations*: LOS indicates length of stay in hospital; NIMV and IMV, noninvasive and invasive mechanical ventilation; ICU, intensive care unit.

* Percentages calculated on patients in the follow-up at 3 years (N = 55 and N = 222 in the group of patients with and without a 30-day readmission respectively).

**Table 6:** The relative risks, absolute relative risks and number needed to harm of the events (mortality in the follow-up period at30 days, 6 months, 1 year and 3 years).

	Estimate of RR [95%CI]	Estimate of ARR [95%CI]	Estimate of NNH [95%CI]
30-day mortality	6.67 [1.14 to 39.07]	-0.04 * [-0.12 to -0.004]	25 † [8 to 243]
6-month mortality	2.80 [1.63 to 4.81]	-0.17 [-0.30 to -0.07]	6 [3 to 14]
1-year mortality	2.13 [1.41 to 3.23]	-0.19 [-0.32 to -0.08]	6 [3 to 13]
3-year mortality	1.56 [1.23 to 1.97]	-0.24 [-0.37 to -0.09]	5 [3 to 11]

* Sample interpretation: in patients with a 30-day readmission, the estimated absolute increase in mortality risk at 30 days was 4%.

^†^ Sample interpretation: for every 25 patients readmitted, one death was caused within 30 days.

*Abbreviations*: RR, relative risk; CI, confidence interval; ARR, absolute relative risk; NNH, number needed to harm.

**Table 7:** General characteristics of patients lost to follow-up (1 year follow-up).

Variables	Patients considered for the study (N = 351)	Patients lost to 1-year follow-up (N = 27)	p value
Age, years	71.4 ± 10.0	71.2 ± 10.0	0.992
Male	297 (85)	21 (78)	0.349
BMI, kg/m^2^	27.4 ± 5.5	27.3 ± 5.4	0.866
Smoking habit: Current/No or Former, %	37/63	67/33	**0.002**
FEV_1_, % predicted	43.7 ± 16.5	51.2 ± 20.2	0.075
FEV_1_/FVC	48.8 ± 14.6	46.3 ± 11.9	0.499
GOLD stage: A/B/C/D, %	3/29/49/19	8/46/27/19	0.071
LTOT	100 (28.5)	4 (14.8)	0.125
mMRC baseline dyspnea grade	2 (1; 3)	1.5 (1; 2)	**0.017**
COPDSS severity questionnaire	14 (9; 19)	10 (5; 14)	**0.007**
Charlson index	2 (1; 3)	2 (1; 3)	0.558
*Use of domiciliary medications*			
Salbutamol only	12 (4)	0 (0)	>0.99
Anticholinergic only	15 (5)	3 (15)	0.087
LABA + Anticholinergic	4 (1)	0 (0)	>0.99
LABA + ICS	8 (3)	1 (5)	0.435
Anticholinergic + ICS	4 (1.3)	1 (5)	0.271
LABA + Anticholinergic + ICS	103 (33)	6 (30)	0.759

*Abbreviations*: see Table 1.

### Predictors of 1-Year Mortality in the Multivariate Analysis

The multivariate Cox regression model with death at one year as a dependent variable (Table 8) showed that a history of two or more exacerbations in the previous year (HR, 3.62; 95% CI, 1.70–7.70), increased dyspnea perception (reflected in an mMRC dyspnea score of 2 or more, HR, 4.71; 95% CI, 1.40–15.82), increased levels of PaCO_2_ (+1 mmHg; HR, 1.02; 95% CI, 1.01–1.04), both evaluated at initial admission for the index hospitalization and at 30-day readmission for AECOPD (HR, 2.48; 95% CI, 1.10–5.59) were independent significant predictors of mortality.

**Table 8:** Significant univariate and multivariate Cox regression analyses predicting the probability of death at 1 year.

Variable	Univariate			Multivariate		
	HR	95% CI	p value	HR	95% CI	p value
Age ≥70 y	2.17	1.26–3.74	0.005	-	-	-
Men	2.13	0.92–4.90	0.077			
BMI *			0.041	-	-	-
Underweight: <18.5 kg/m^2^	1.07	0.25–4.56	0.92	-	-	-
Normal weight: ≥18.5 and <25 kg/m^2^	1	-	-	-	-	-
Pre-obese: ≥25 and <30 kg/m^2^	0.42	0.21–0.84	0.014	-	-	-
Obese: ≥30 kg/m^2^	0.44	0.20–0.96	0.039	-	-	-
Smoking habit †			<0.001	-	-	-
No	1	-	-	-	-	-
Current	0.25	0.07–0.91	0.035	-	-	-
Former	0.94	0.30–3.02	0.92	-	-	-
Temperature (+1°C)	0.75	0.54–1.03	0.071	-	-	-
No of exacerbations ≥2 ‡	2.26	1.38–3.69	0.001	3.62	1.70–7.70	0.007
No of exacerbations requiring hospitalization ≥2 ‡	2.61	1.51–4.52	0.001	-	-	-
COPD-SS ≥15 §	4.22	2.47–7.21	<0.001	-	-	-
Charlson index ≥3 §	1.66	1.04–2.66	0.034	-	-	-
mMRC dyspnea score ≥ 2 §	5.22	1.84–14.81	0.002	4.71	1.40–15.82	0.012
FEV_1_ <50% **	3.29	1.56–6.93	0.002	-	-	-
FVC <50% **	2.09	1.16–3.78	0.015	-	-	-
Haematocrit (+1%) §	0.96	0.92–1.00	0.067	-	-	-
Haemoglobin (+1 gr/L) §	0.99	0.98–1.00	0.030	-	-	-
pH (+0.1 units) §	0.70	0.52–0.93	0.015	-	-	-
PaCO_2_ (+1 mmHg) §	1.02	1.01–1.03	<0.001	1.02	1.01–1.04	0.005
30-day readmission for AECOPD	2.58	1.57–4.24	<0.001	2.48	1.10–5.59	0.029
LOS (+1 day)	1.06	1.04–1.09	<0.001	-	-	-

* The p-value corresponds to differences between the four groups (underweight: <18.5 kg/m^2^, normal weight: ≥18.5 and <25 kg/m^2^, pre-obese: ≥25 and <30 kg/m^2^, or obese: ≥30 kg/m^2^).

^†^ The p-value corresponds to differences between the three groups (non-smoker, current smoker, or former smoker).

^‡^ In a period of 1 year prior to index hospitalization.

^§^ Evaluated at admission for index hospitalization.

** Data evaluated at least 6 months prior to index hospitalization.

*Abbreviations*: see Tables 1, 2 and 5.

## Discussion

Our study found that COPD patients with an acute exacerbation requiring hospitalization within 30 days of discharge have a subsequent progressive increase in the risk of death over three years of follow-up, independently of the severity of airflow obstruction. Moreover, a history of two or more exacerbations in the previous year predicts readmission within a month of discharge.

In the comparison of baseline characteristics between patients with and without a 30-day readmission for AECOPD, several variables differed between the two groups (lower predicted FEV_1_%, greater prevalence of GOLD stages C and D, greater prevalence of LTOT, higher perceived level of dyspnea [mMRC score], and a higher score on the COPD severity score questionnaire). In addition, we found that two or more prior AECOPD recorded in the year prior to the index event was an independent predictor of a new readmission within 30 days of discharge. This finding, corroborate the hypothesis of a specific susceptibility to exacerbation in some COPD patients [5,6], reported in previous studies which showed that a history of previous events was the most important predictor of subsequent exacerbations [13,21]. As reported elsewhere [13], microbiological and early inflammatory patterns at admission did, not differ and did not affect the likelihood of AECOPD within 30 days of discharge.

With regard to outcomes, hospital length of stay (LOS), was clearly higher in severe readmitted patients, but it was interesting to see that the use of NIMV and IMV and the number of ICU admissions were comparable between groups. This confirms the similar degree of AECOPD severity at the index hospitalization. In our short and long-term evaluation, however, 30-day readmission for AECOPD predicts a subsequent, progressive increase in the risk of death for all causes (Tables 5 and 6, Fig 2); in terms of prognosis in patients hospitalized for AECOPD, our findings may support the hypothesis of a frequent exacerbator phenotype of COPD [5,6] which would be of particular relevance in both short and long-term management and that needing “*ad hoc”* treatments (use of chronic antibiotics therapy, early rehabilitation program) or monitoring visit (few days after discharge)Moreover, with regard to the relationship between frequency of AECOPD and mortality in COPD patients, our results corroborate those of two previous studies published in 2005 [8] and 2014 [21]. In a prospective cohort of 304 stable COPD patients followed up for a period of five years, Soler-Cataluña et al. [8] found that patients with three or more exacerbations in the initial year had an increased risk of mortality due to all causes in comparison to patients with two exacerbations or fewer; similarly, in a 4-year secondary data analysis of Italian health care administrative databases comprising more than 15,000 COPD patients, Blasi et al. [21] found that patients with at least one severe AECOPD within three years prior to the index period had a higher mortality risk than patients with only moderate or with no episodes of AECOPD. However, neither of those studies report data on the mortality of patients with early readmission (i.e., within 30 days of hospital discharge).

Although readmission within 30 days for AECOPD was an independent significant risk factor in our multivariate model predicting survival at 1 year (Table 8), in agreement with other studies [8,22,23] we found other clinical risk factors such as higher levels of dyspnea perception [22] (related to a greater lung impairment) and hypercapnia [8,23] (suggestive of chronic alveolar hypoventilation and reflecting the severity of the underlying respiratory condition) to be negative predictors of mortality. In addition, although it has been demonstrated that co-morbidities have an impact on the short-term and medium-term outcome of AECOPD [24] and although a previous study [13] found diabetes to be a significant predictor of early readmission, we found no correlations or role of co-morbidities in the prognosis of AECOPD. The incomplete recording of all co-morbidities linked to COPD together may explain this difference [13].

Major strengths of our research are the prospective and consecutive nature of the data collection, the large cohort of patients enrolled in the long-term follow-up, the statistical approach, which allows the first evaluation of NNH with readmission as an exposure risk, and the exclusion of patients with CAP and heart failure.

This study has some limitations. First, it was conducted at a single centre, and so the extrapolation of these findings to other settings must be done with care. Second, we lack information on the cause of death and on the systematic analysis of serum inflammatory biomarkers during the pre-index stable phase of COPD, at discharge, and at each follow-up visit. Third, no adjustments were made for multiple comparisons. Finally, we cannot rule out the possibility that a relapse of the initial AECOPD may have been the cause of readmission; as just noted, the lack of data on serum inflammatory levels at discharge and in the follow-up visit at 30 days in non-resolving exacerbations [25] did not allow us to confirm our clinical evaluation.

## Conclusion

In hospitalized AECOPD patients, readmission due to an acute exacerbation within 30 days of discharge is associated with a progressive increase in the long-term risk of death. Moreover, a history of ≥2 previous exacerbations predicts the 30-day readmission of COPD patients.
